# Whole genome molecular analysis of respiratory syncytial virus pre and during the Covid-19 pandemic in free state province, South Africa

**DOI:** 10.1016/j.virusres.2024.199421

**Published:** 2024-07-05

**Authors:** Hlengiwe Sondlane, Ayodeji Ogunbayo, Celeste Donato, Milton Mogotsi, Mathew Esona, Ute Hallbauer, Phillip Bester, Dominique Goedhals, Martin Nyaga

**Affiliations:** a Next Generation Sequencing Unit and Division of Virology, Faculty of Health Sciences, University of the Free State, Bloemfontein, South Africa; b Enteric Diseases Group, Murdoch Children's Research Institute, Parkville, VIC, Australia; c Department of Paediatrics, The University of Melbourne, Parkville, VIC, Australia; d The Centre for Pathogen Genomics, The Doherty Institute, University of Melbourne, Australia; e Diarrheal Pathogens Research Unit, Sefako Makgatho Health Sciences University, Medunsa 0204, Pretoria, South Africa; f Department of Paediatrics and Child Health, Faculty of Health Sciences, University of the Free State, Bloemfontein, South Africa; g Division of Virology, School of Pathology, Faculty of Health Sciences, University of the Free State, Bloemfontein, South Africa; h PathCare, Pretoria, South Africa

**Keywords:** Respiratory syncytial virus, COVID-19 pandemic, Whole genome sequencing

## Abstract

Respiratory syncytial virus (RSV) is the most predominant viral pathogen worldwide in children with lower respiratory tract infections. The Coronavirus disease 2019 (COVID-19) pandemic and resulting nonpharmaceutical interventions perturbed the transmission pattern of respiratory pathogens in South Africa. A seasonality shift and RSV resurgence was observed in 2020 and 2021, with several infected children observed. Conventional RSV-positive nasopharyngeal swabs were collected from various hospitals in the Free State province, Bloemfontein, South Africa, from children suffering from respiratory distress and severe acute respiratory infection between 2020 to 2021. Overlapping genome fragments were amplified and complete genomes were sequenced using the Illumina MiSeq platform. Maximum likelihood phylogenetic and evolutionary analysis were performed on both RSV-A/-B G-genes with published reference sequences from GISAID and GenBank. Our study strains belonged to the RSV-A GA2.3.2 and RSV-B GB5.0.5a clades. The upsurge of RSV was due to pre-existing strains that predominated in South Africa and circulating globally also driving these off-season RSV outbreaks during the COVID-19 pandemic. The variants responsible for the resurgence were phylogenetically related to pre-pandemic strains and could have contributed to the immune debt resulting from pandemic imposed restrictions. The deviation of the RSV season from the usual pattern affected by the COVID-19 pandemic highlights the need for ongoing genomic surveillance and the identification of genetic variants to prevent unforeseen outbreaks in the future.

## Highlights

• The COVID-19 pandemic caused significant disruptions to the prevalence and spread of respiratory syncytial virus (RSV) and other respiratory viral infections in South Africa during the period of 2020-2021.
• The surge in respiratory syncytial virus (RSV) infections among children under the age of 5 has been attributed to the COVID-19 lockdown pressure due to extended non exposure to RSV.
• During the pandemic, using masks and social distancing reduced RSV transmission, leading to a population with reduced immunity, known as 'immunity debt'.
• Phylogenetic studies on global RSV evolution are hindered by limited access to complete genome sequences deposited in public databases.
• Genetic variation in RSV in South Africa is poorly studied, as is the transmission and dissemination of RSV within different communities and the duration of individual RSV lineages persisting in a population.

## 1 Introduction

Respiratory syncytial virus (RSV) is recognised as the most frequent cause of viral lower respiratory tract infection (LRTI) in infants and children ≤5 years worldwide, and a leading cause of hospitalisation, mortality, and morbidity and (Demont et al., 2021; Shi et al., 2017; Weinberg, 2017). The RSV infects nearly infection is 90 % of children within the first two years of life, with nearly 40 % of whom develop LRTI the first infection episode (Brady et al., 2014; Meissner, 2016). Also, RSV offers partial immunity due to waning immunological memory, characterized by recurrent infections that may continue into adulthood (Griffiths et al., 2017; Nam and Ison, 2019). Almost 90 % of RSV-associated fatalities are recorded in children ≤5 years from low income (or low resourced) countries (Liu et al., 2020).

In temperate regions, RSV has a distinct seasonal circulation in the late autumn or early winter and tropical regions infections predominate during the rainy season (Khor et al., 2012). Despite the global impact of RSV on public health, there are currently no licensed RSV vaccines or effective antivirals for acute infection available (Feng et al., 2022; Mazur et al., 2018). Palivizumab prophylactic monoclonal antibody (mAb) has been widely used in the prevention of RSV and has proven to be effective against RSV infections in high-risk children (Cadena-Cruz et al., 2023; Elawar et al., 2021). In addition, novel mAb Nirsevimab, a newly approved by the FDA for the prevention of RSV infection in infants (Simões et al., 2023). Another vaccine, known as ABRYSVO, administered in pregnant women, to protect infants from RSV associated LRTI after birth and individuals who are 60 years old and above (Ernst, 2023).

Recently RSV has been reclassified under the genus human *orthopneumovirus*, belonging to the family *Pneumoviridae* and order monogavirales (Rima et al., 2017). The genome has approximately 15, 200 nucleotides of single stranded, negative sense RNA encoding 11 viral proteins (Azzari et al., 2021). The virus is classified into RSV-A and RSV-B, the two primary antigenic groups that have been identified as the prevalent strains within the human population (Vandini et al., 2017). The attachment glycoprotein (G) has been extensively employed for RSV genotyping due to its high degree of diversity (Goya et al., 2020; Hibino et al., 2018; Muñoz-Escalante et al., 2021). Numerous studies have proposed a unified manner of designating genotypes. Adoption and the implementation of this methodology facilitated the categorization of lineages and sub-genotypes or clades, while simultaneously decreasing the number of identifiable genotypes by almost half in both groups (Chen et al., 2022; Goya et al., 2020). Based on the newly proposed genotype nomenclature, three genotypes were identified and classified within RSV-A, namely GA1, GA2, and GA3, similarly, within RSV-B, six genotypes were designated as GB1, GB2, GB3, GB4, GB5, GB6 and GB7 (Goya et al., 2020).

Before the COVID-19 pandemic, an estimated 6,000 RSV-associated deaths occurred annually in South Africa (Cohen et al., 2018). The unprecedented COVID-19 pandemic prompted the South African government and other nations to implement nonpharmaceutical interventions (NPIs) to contain the spread of SARS-CoV-2. These NPIs included social distancing, frequent hand washing, travel restrictions, school closures, curfews, and mask wearing (Alanezi et al., 2020; Bents et al., 2022; Ijaz et al., 2022). Although the implemented NPIs were successful in controlling SARS-CoV-2, they have indirectly disrupted the circulation of other respiratory viruses with a known seasonal pattern, such as RSV, in many countries worldwide (Baker et al., 2020; Tempia et al., 2021; Yeoh et al., 2021; Zheng et al., 2021). Surveillance data from South Africa suggested a significant decline in RSV circulation from the 2020-2021 winter season (Olsen et al., 2021; Tempia et al., 2021). The impact of the NPIs was not uniform amongst the respiratory pathogens, but there was a simultaneous reduction of influenza, and RSV during the COVID-19 pandemic compared to Rhinoviruses and respiratory Adenoviruses (Abo et al., 2021; Tempia et al., 2021). A delayed RSV season was observed in late 2020 and early 2021 and its resurgence was accompanied by offseason outbreaks following the gradual relaxation of lockdown restrictions (Agha and Avner, 2021; van Summeren et al., 2021). The altered epidemiology and resurgence of RSV was probably due to waned immunity from reduced or lack of exposure to the virus, (Reicherz et al., 2022). This highlights the importance of molecular surveillance to predict potential outbreaks that could have an impact on vulnerable populations.

There is limited data on characterisation of strains driving the RSV epidemic in South Africa, and none in the Free State province. Currently, there is a dearth of whole genome data on RSV strains in public databases, particularly during the COVID-19 era in South Africa. Next generation sequencing technology and its applicability, allow the broader characterisation of circulating RSV strains through whole genome sequencing (WGS), and this information could aid in antiviral medication and vaccine development using epidemiologic surveillance and characterisation of RSV diversity (Wang et al., 2022). The progress made in the field of WGS presents a significant opportunity to tackle unresolved concerns related to the viral evolution of RSV and reduce dearth of information in underrepresented communities and less described sequenced genomic regions of RSV (Rios Guzman and Hultquist, 2022).

To the best of our knowledge, this is the first study to describe RSV complete genome sequences collected in South Africa during the COVID-19 pandemic. However, it is unclear whether the RSV outbreaks observed in this period were due to the emergence of novel strains with an enhanced selective advantage or influenced by selective pressure resulting from the low RSV transmission in 2020. The objective of this study was to perform whole genome sequencing of RSV in children ≤5 years of age with respiratory distress and severe acute respiratory illness (SARI) and investigate circulation patterns of RSV during the COVID-19 pandemic in South Africa.

## 2 Methodology

### 2.1 Study design and patient enrolment

This study utilised samples collected from two existing studies that previously received ethical clearance. Samples were utilised from a paediatric clinical study enrolling children (0-12 years) with respiratory distress requiring hospital admission at Pelonomi Regional Hospital, Universitas Academic Hospital, or National District Hospital in the Free State Province, South Africa. Nasopharyngeal swabs (NPs) were collected and transported for routine testing of RSV, SARS-CoV-2, and *Bordetella pertussis* at the National Health Laboratory Services (NHLs), Bloemfontein, Free State Province, South Africa. Secondly, the cross-sectional study recruited children who were presenting with severe acute respiratory tract infection (SARI) and required hospitalisation. Patients were recruited in Botshabelo District Hospital, Pelonomi Regional Hospital, and National District Hospital in the Free State Province, South Africa. The World Health Organisation (WHO) definition of SARI was used as criteria for sample collection as described previously (Ogunbayo et al., 2022). Nasopharyngeal swabs (BD Diagnostics, Franklin Lakes, NJ, USA) were collected from the children and inserted into a viral transport media (VTM) and placed in ice (4 ℃) then transported to the University of the Free State Next Generation Sequencing Unit (UFS-NGS Unit and were stored at -80 °C until processed. Subsequently, the NPs were tested for several respiratory viruses including RSV as described previously (Ogunbayo et al., 2022). Upon admission, clinicians and or nurses collected relevant clinical and demographic information. Study numbers were used instead of patient identification information.

### 2.2 Ethical consideration

The study protocol was approved by the Health Science Research Ethics Committee (HSREC) of the University of the Free State (HSREC initial approval number: UFS-HSD2021/1616/2501). The Environmental and Biosafety Ethics Research Committee also granted clearance for the use of biological samples in this study (EBREC number: UFS-ESD2021/0256/21). Furthermore, permission to acquire/retrieve samples at NHLS was granted by the Academic Affairs Research Management System (AARMS). Additional HSREC approval was sought, for the additional samples used in this study (HSREC number: (UFS-HSD2021/1616/2501-0002). All procedures were carried out in compliance with the established institutional guidelines.

### 2.3 Sample collection

Data and paediatric cases were collected July 2020 to July 2021 from children in the Free State province. The NPs that were identified as positive for RSV were selected in children ≤5 years at the time of sampling and samples collected during the COVID-19 pandemic. These samples were retrieved at NHLs (n=50) and UFS-NGS unit (n=19) archives along with the provision of cycle threshold (Ct) values and the list of detected viruses on respiratory panels.

### 2.4 RNA extraction and cDNA synthesis

Viral RNA was extracted directly from 250 μL of RSV-positive clinical sample using the QIAamp Viral RNA mini kit (Qiagen, Hilden, Germany), according to the manufacturer's recommendations, except for the use of a carrier RNA. RNA eluted in 50 μL of elution buffer was stored at -20 ℃ until use. Additionally, samples with low-quality RNA were further extracted using an automated extraction machine Chemagic^Tm^ 360 (PerkinElmer, Waltham, MA, USA (PerkinElmer), according to the manufacturer's recommendations.

Subsequently, the RNA was reverse transcribed to cDNA and four overlapping RSV fragments were simultaneously amplified in an independent reaction using the SuperScript IV One-Step RT-PCR System (Thermo Fisher Scientific, Waltham, MA, USA) as previously described (Langedijk et al., 2020). The PCR fragments were resolved by agarose gel electrophoresis (2 % agarose gel) and visualised using a gel imaging system, then quantified using Qubit^TM^ 1X dsDNA High sensitivity assay kit (Thermo Fisher, Waltham, MA, USA), normalised and pooled in equimolar amounts per amplicon for each sample. Finally, the amplified cDNA products were purified using Wizard® SV Gel and PCR Clean-Up System (Promega, Wisconsin, USA), according to the manufacturer's instructions.

### 2.5 Whole genome sequencing of RSV

The purified amplicons were used as a template for Illumina NGS library preparation using the QIAseq FX single cell RNA library preparation kit (Qiagen, Hilden, Germany), following the manufacturers instruction. Prior to sequencing, a Bioanalyzer Agilent 2100 instrument (Agilent Technologies, Santa Clara, CA, USA), was used to analyse the library fragment size distribution. The libraries were then normalized using Qubit 2.0 (Thermo Fisher, Waltham, MA, USA), pooled, diluted, and sequenced using 300-cycles MiSeq reagent v2 standard kit (2×150 bp paired-end) (Illumina, San Diego, CA, USA).

### 2.6 Data analysis

#### 2.6.1 Genome assembly

Raw pair-end sequence reads were analysed using Genome Detective, an online based bioinformatics tool (https://www.genomedetective.com/, Accessed 22 November 2022) (Vilsker et al., 2019). The RSV subtypes were assigned, and genome coverage was generated for each sample during the assembly process. Sequences with over 99 % genome coverage were further inspected and analysed using Geneious Prime® version 2022.0.1 (Kearse et al., 2012). The reads were assembled/mapped to a reference sequence for both RSVA and B (Accession number NC_013235 and NC_ 001781), respectively using Geneious Prime®, and generated a consensus sequence for each sample.

#### 2.6.2 Generation of global datasets for phylogenetic analysis

All global RSV samples available in the GISAID database (Shu and McCauley, 2017), were downloaded (accessed 24 June 2023). Additionally, all RSV strains from the African continent available in the Bacterial and Viral Bioinformatics Resource Center (BV-BRC, https://www.bv-brc.org/) (Olson et al., 2023), were also downloaded. The Kenyan strains from BV-BRC were excluded due to the overrepresentation of Kenyan strains in the dataset and the GISAID Kenyan dataset was stratified by year and identical sequences removed. Identical sequences representing the repeated sequencing of the same isolate (determined by the corresponding isolate name and date of collection) were removed where identified.

Separate RSV A and B datasets were generated, and multiple sequence alignments were performed using MAFFT v7.490 (Katoh et al., 2002; Katoh and Standley, 2013), applying the FFT-NS-2 algorithm in Geneious. Visually inspected whole genome alignments of RSV A and B were trimmed to the open reading frame of the G comprising of 963 bp and 1722 bp of F genes. Strains with poor sequencing quality, shorter than 300 bp or lacking sampling date or isolation country were excluded.

Prior to phylogenetic analysis, the RSV A and B alignments were tested for recombination by applying multiple models (RDP, GENECONV, Bootscan, Chimaera, SiScan, MaxChi, and 3Seq) using Recombination Detection Program version 5 (RDP5) and any strains with recombination events detected by three or more models were removed from the dataset (Martin et al., 2021).

#### 2.6.3 Recombination

Potential signals for genomic inter-subgroup recombination events were investigated in samples sequenced in this study except for sample hRSV/A/ZAF/UFS-NGS-UNIT/RD-3|2021-02-24 which was excluded due to poor sequence quality. The consensus of the RSV/A samples was used as the comparison reference genome in a similarity plot analysis. A Jukes-Cantor distance model was applied with a window length of 200 bp and step size of 20 bp applied within Simplot 3.5 (Samson et al., 2022).

### 2.7 Phylogenetic analysis of RSVA/B strains

#### 2.7.1 Clade assignment

All strains from this study were analysed for clade determination using the online RSV clade typing tool available in Nextclade (https://clades.nextstrain.org) (Aksamentov et al., 2021). Generated phylogenetic trees comprising study strains and reference strains were exported and subsequently visualised using Auspice (https://auspice.us) (Hadfield et al., 2018).

#### 2.7.2 Maximum likelihood phylogenetic tree analysis

All global strains were classified into G-clade using the RSV clade typing tool available in Nextclade (https://clades.nextstrain.org) and randomly subsampled, clade-specific alignments were generated for GB5.0.5a and GA2.3.5. The study strains under investigation were derived from complete whole genome sequences, with the complete G-gene extracted comprising of 11 RSV-A and 9 RSV-B genomes. Additionally, a total of 267 RSV-A and 118 RSV-B South African reference strains were used for phylogenetic analysis, derived from either whole genome, partial genome, or the complete G-Gene. The optimal nucleotide substitution model for each dataset was determined using ModelFinder (Kalyaanamoorthy et al., 2017). Maximum likelihood (ML) phylogenetic trees were inferred using IQTree version 1.6.12 and node support values were evaluated by bootstrapping to 1000 replicates (Nguyen et al., 2018).

#### 2.7.3 Bayesian evolutionary analysis

G-clade datasets were examined for adequate temporal signal using TempEst and divergent samples were removed (Rambaut et al., 2016). Phylogenetic relationships and viral demographic histories were inferred using BEAST 1.10 (Suchard et al., 2018). The optimal nucleotide substitution model was determined using ModelFinder in IQTree. An uncorrelated relaxed molecular clock was specified with branch rates drawn from a lognormal distribution to account for varied evolutionary rates among lineages. A Gaussian Markov Random Fields (GMRF) Bayesian Skyride demographic prior was specified (Suchard et al., 2018). The chain length of MCMC sampling was 100 million generations with sampling every 10,000 generations. Convergence and mixing were examined using Tracer 1.7 and maximum clade credibility (MCC) trees were summarised using TreeAnnotator.

#### 2.7.4 Selection pressure analysis

Selection pressures acting on the G and F genes were investigated using the Single-Likelihood Ancestor Counting (SLAC), Fixed Effects Likelihood (FEL), (Mixed Effects Model of Evolution (MEME), and Fast, Unconstrained Bayesian AppRoximation (FUBAR) methods (P < .05 or posterior threshold 0.9 to minimize false positives) (Weaver et al., 2018), implemented in HyPhy (Hypothesis Testing using Phylogenies) (version 2.5.33) (Kosakovsky Pond et al., 2020). As results can differ depending on the chosen method, sites selected by three or more methods were considered robust.

#### 2.7.5 N-linked glycosylation

The F and G genes of the RSV A and B datasets comprised of African strains were interrogated to identify putative N-glycosylation sites utilising the N-Glycosite web tool (https://www.hiv.lanl.gov/content/sequence/GLYCOSITE/glycosite.html) (Zhang et al., 2004).

## 3 Results

### 3.1 Patient demographics and clinical characteristics

Participants were enrolled in 2020 to 2021 and a total of 69 samples were collected. The demographic profiles among the participants were similar, comparable, and unbiased in both groups with respiratory distress and SARI. Among the RSV positive patients, 47/69 (68 %) had LRTI, and 11/69 (16 %) had URTI. The remaining 16 % had no clinical data available. The clinical characteristics of children are shown in Table 1.1. Detailed demographics of the participants of which samples were subsequently used in the analysis are represented in the supplementary material Table 1S1-1S2.

**Table 1.1:** Clinical characteristics of patients infected with RSV during the COVID-19 pandemic.

Patient demographics	Paediatric clinical study	Viral metagenomic cross sectional study
**RSV infection cases**	66 % ≤1 year	37 % ≤ 1year
**Gender** **Males** **Females** **Unspecified**	48 % (24/50) 36 % (18/50) 16 % (8/50)	63 % (12/19) 37 % (7/19) -
**Suspected diagnosis** **LRTI** **URTI** **Unspecified**	78 % (39/50) 2 % (1/50) 16 % (8/50)	37 % (7/19) 47 % (9/19) 16 % (3/19)
**PICU admission** **Unspecified**	8 % (4/50) 16 % (8/50)	None 5 % (1/19)
**Feeding difficulty**	-	32 % (6/19)
**HIV infected** **Uninfected** **Exposed** **Unspecified**	4 % (2/50) 58 % (29/50) 22 % (11/50) 16 % (8/50)	11 % (2/19) 89 % (17/19) - -
**Chest indrawing**	-	47 % (9/19)
**Household member smoking** **Unspecified** **Unknown**	16 % (8/50) 16 % (8/50) 2 % (1/50)	16 % (/19) - -

### 3.2 Genome sequencing and assembly

During the study period, only 50 patients with respiratory distress and 19 with SARI were RSV positive. Of these, 72 % (50/69) samples had quantifiable viral RNA extracted, of which 76 % (38/50) were amplified, and 95 % (36/38) were sequenced. In this group 58 % (21/36) of the samples had RSV whole genomes successfully sequenced [17 ≥ 99 % genome coverage, while four were near-complete (approx. 80 % genome coverage)]. Of the total sequenced samples 25/36 were identified as subtype RSV-A and 11 as subtype RSV-B with genome coverage ranging from 80-100 % (Supplementary Table 1S3-1S4)

### 3.3 Recombination analysis on sequenced study strains

**Fig. 1.1:**
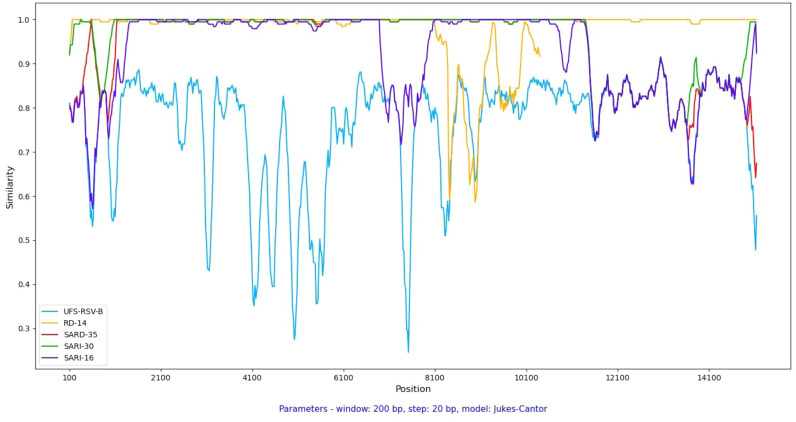
Recombination analysis of the complete genome of the sequenced study strains. The consensus of non-recombinant RSV-A study strains was used as the reference whilst the consensus of RSV/B study strains is presented by “UFS-RSV-B”.

Four RSV-A study strains were identified as potential inter-subtype recombinants with multiple breakpoints across the genome and regions derived from RSV-B (Fig. 1.1, Supplementary Table 1S5).

### 3.4 South African RSV positive cases before and during the COVID-19 pandemic

**Fig. 1.2:**
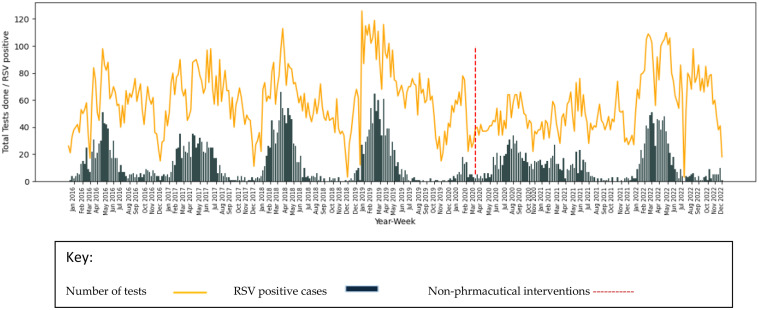
Depiction of RSV epidemiology in South Africa. The key indicate the number of specimens tested and positive tests by year/week among children below 5 years over the period of 2016–2022. The red dotted line marks and represents the beginning of NPI's impelmentation in South Africa.

In South Africa, the first case of COVID-19 was reported on March 5, 2020, and a nationwide lockdown was subsequently imposed on March 27, 2020. The implementation of non-pharmaceutical interventions during the COVID-19 pandemic, such as travel restrictions, mask-wearing, social distancing, and lockdowns, had initially led to a decrease in the transmission of various respiratory viruses including RSV. Following the relaxation of the implemented NPI's, there were notable summer outbreaks of RSV in South Africa in late 2020. The epidemiology of RSV before and during the COVID-19 pandemic in South Africa, data on RSV positive cases from both inpatients and outpatients at the national institute of communicable diseases (NICD) were examined from January 2016 to December 2022 (Fig. 1.2). Prior to 2020, RSV activity typically began in mid-autumn and persisted throughout the winter months with a peak in cases during winter. However, the easing of these restrictions resulted in a resurgence of RSV cases, which had been relatively low for over a year. During the COVID-19 pandemic, the absence of RSV during winter led to the emergence offseason outbreaks, marking a significant shift from the usual seasonal distribution of the virus. This is evident from the laboratory-confirmed RSV positive cases reported in 2020, which were associated with an offseason outbreak in summer (Fig. 1.2).

### 3.5 Clade assignment

**Fig. 1.3:**
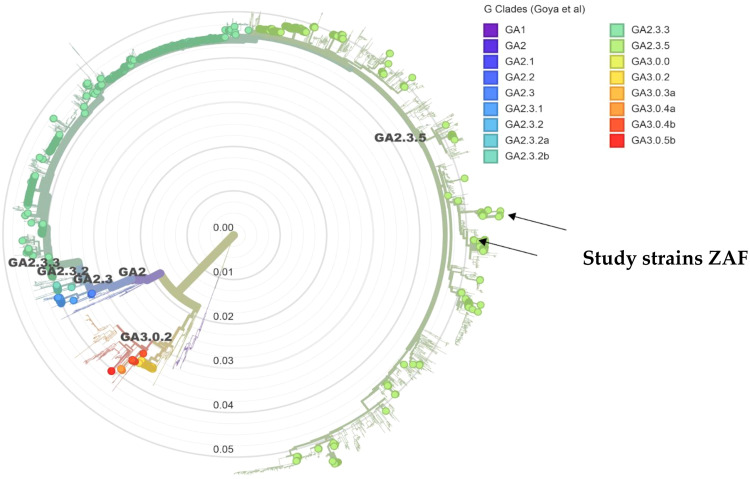
Phylogenetic tree of major RSV-A clades generated using NextClade. Previously characterised South African taxa are represented by circles coloured based on clade. The strains from this study formed two separate clusters (indicated with black arrows) within GA2 clade GA2.3.5 sub-genotype.

**Fig. 1.4:**
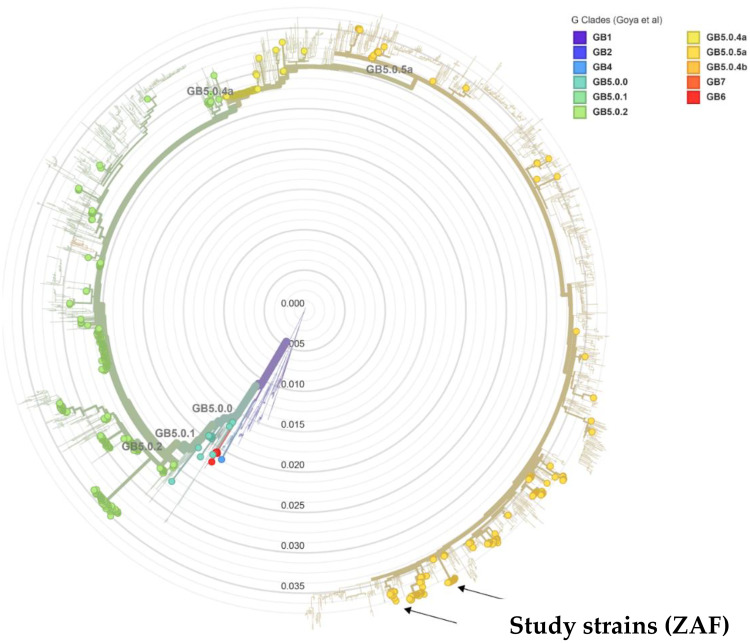
Phylogenetic tree of major RSV-B clades generated using NextClade. Previously characterised South African taxa are represented by circles coloured based on clade. The strains from this study formed two separate clusters (indicated with black arrows) within GB5 clade GB5.0.5a sub-genotype.

The RSV-A strains from this study were classified as clade GA2, corresponding to the previously recognised ON1 genotype. Six lineages have been identified within the GA2.3 sub-genotype (GA2.3.1‐GA2.3.6) with strains from this study clustering within GA2.3.5 (Fig. 1.3). The RSV-B strains were classified within clade GB5.5 corresponding to the previously recognised BA type. Five lineages have been identified within GB5.0 (GB5.0.1‐GB5.0.5). The samples from this study belong to GB5.0.5a (Fig. 1.4).

### 3.6 Maximum likelihood phylogenetic tree of RSV-A

**Fig. 1.5:**
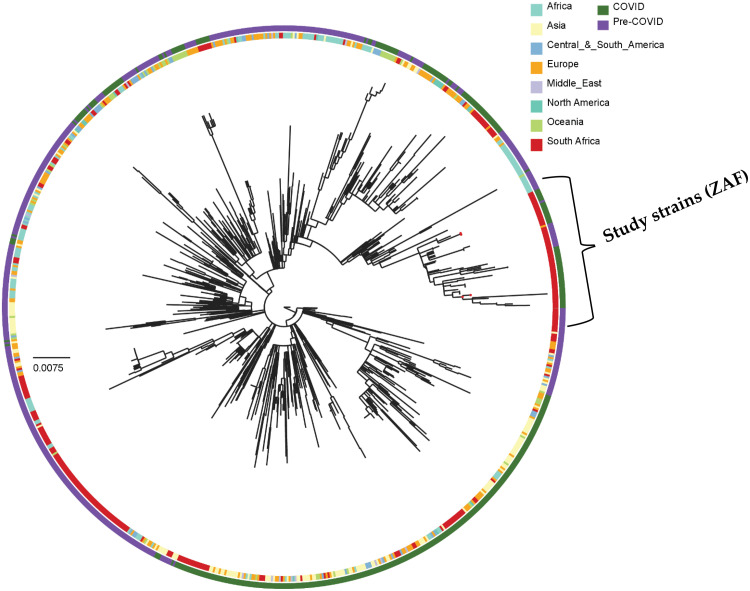
Phylogenetic tree of global representative GA2.3.5 strains constructed by maximum likelihood method at 10000 bootstrap replicates. Study strains are indicated by a red circle. The bootstrap values are not displayed at the branch nodes of the tree. A detailed phylogenetic tree with bootstrap support shown is presented in Supplementary Figure 1S1. The phylogenetic tree is drawn to scale; the scale bar represents the number of nucleotide substitutions per site.

In the pre-COVID-19 era, specifically, from 2015 to 2018, there were several co-circulating variants of GA2.3.5 in South Africa, showing high genetic similarity to global strains, primarily from Europe, North America, and other countries within the African continent (Fig. 1.5). The phylogenetic tree showed minor endemic circulation of strains within Africa reflecting the continued introduction of variants from outside the continent. Several variants that circulated in in the pre-COVID-19 era were not detected from 2018 onwards including the dominant South African variant that circulated between 2015-2018 which was also detected in Mozambique.

The strains detected in South Africa between 2020-2021 were genetically diverse forming a number of small clusters and most of these small clusters were closely related to South African strains detected in 2018, suggesting that the increase in RSV-A detected was not due to a dominant new variant recently introduced into South Africa, rather the population was largely derived of strains that had likely evolved within the country over the intervening years. A small number of variants appeared to be recent introductions following the re-opening of borders showing a high degree of genetic similarity to globally circulating strains. No unique clustering patterns were observed with strains from other regions that could suggest sustained introductions of novel variants into the population. The samples from the Free State formed two discrete clusters, with the largest cluster forming a monophyletic cluster with contemporary South African strains from other provinces and most closely related to samples from the Gauteng province. Based on the phylogeny this variant was likely derived from viruses that circulated in Kenya between 2015-2018. There was a high degree of circulation of all variants across the country indicating the widespread nationwide transmission of variants.

### 3.7 Maximum likelihood phylogenetic tree of RSV-B

**Fig. 1.6:**
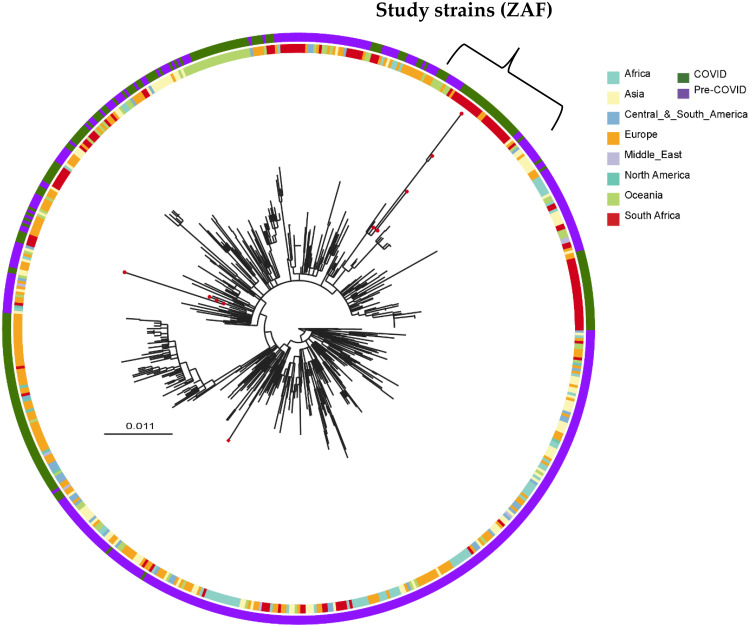
Maximum likelihood phylogenetic tree Global G-Gene GB5.0.5a strains. Study strains sequenced in this study are shown in a red circle. A detailed phylogenetic tree with bootstrap support shown is presented in Supplementary Figure 1S2. The scale number indicates the number of nucleotide substitutions per site.

The sequencing data for South African RSV-B strains indicated that GB5.0.5a circulated as the dominant variant from the mid-2000s to 2015 with no sequencing data from 2016 and 2017. The GB5.0.5a was a minor variant in 2015 and emerged in 2018 to become the dominant clade (Fig. 1.6). Between 2018 and 2019, multiple GB5.0.5a variants co-circulated within South Africa (Fig. 1.6). These strains exhibited a high degree of genetic diversity, forming numerous clusters with contemporary strains of diverse geographic origins reflecting the repeated seeding of global variants into South Africa rather than the endemic circulation of African variants. The dominant lineage of South African strain that circulated prior to the COVID-19 era was largely undetected after 2020. Between 2020 and 2022, multiple GB5.0.5a variants co-circulated with contemporaneous strains from various geographic regions with the strains probably introduced recently into South Africa rather than evolving from variants which circulated in the country prior to the COVID-19 era. The samples from the Free State formed two discrete clusters, with the largest cluster forming a monophyletic cluster with contemporary South African strains from other provinces and most closely related to samples from the Western Cape and North West provinces. The smaller Free State cluster was not closely related to contemporary South African strains and were most closely related to strains from various geographic regions. There was a high degree of circulation of all variants across the country with limited geographic clustering indicating the widespread nation-wide transmission of variants.

### 3.8 Bayesian reconstruction of evolutionary histories

The time to most recent common ancestor (TMRCA) of the South African GA2.3.5 strains from the most recent tip (22nd of June 2022) (Figure 1S3), was 11.64 years (95 % HPD interval [11.0216, 12.2639]) resulting in an estimated emergence of this clade in late 2010 (2010.833, 95 % HPD interval [2010.2074, 2011.4497]). The nucleotide substitution rate for the South African GA2.3.5 strains was estimated at 2.033× 10^−3^ (95 % HPD interval [1.6708× 10^−3^, 2.428× 10^−3^]. The TMRCA of the South African GB5.0.5a strains from the most recent tip (19th of July 2022) was 8.51 years (95 % HPD interval [7.8568, 9.2842]) resulting in an estimated emergence of this clade in early 2014 (2014.035, 95 % HPD interval [2013.261, 2014.6884]). The nucleotide substitution rate for the South African GB5.0.5a (Figure 1S4), strains was estimated at 4.081 × 10^−3^ (95 % HPD interval [2.5524× 10^−3^, 5.801× 10^−3^]).

### 3.9 The relative genetic diversity of the GA2.3.5 and GB5.0.5a strains

**Fig. 1.7:**
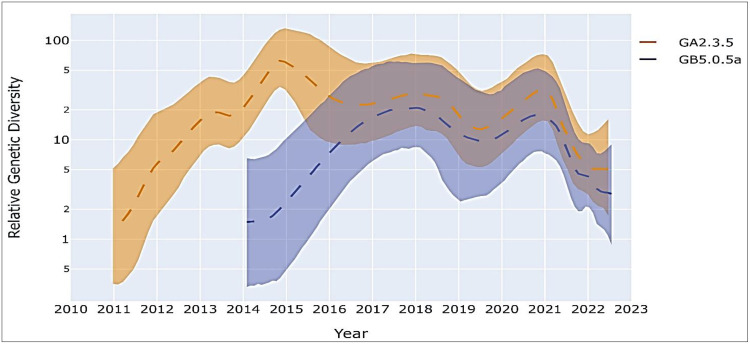
The relative genetic diversity of South African GA2.3.5 and GB5.0.5a strains. A measure of relative genetic diversity is given on the y-axis with the 95 % highest posterior density shown in solid colour and the median as a dashed line.

Between 2017 and 2022 the South African GA2.3.5 and GB5.0.5a strains showed very similar patterns of genetic diversity with a slight bottleneck observed between 2019 and 2020 which represented multiple variants ceasing to circulate in the population during the early years of the COVID-19 era coinciding with lockdowns and extensive NPIs. This was followed by an increase in diversity between 2021 and 2022 as multiple variants co-circulated with the introduction of new variants, with diversity decreasing throughout 2022 due to reduced sampling (Fig. 1.7)

### 3.10 Selection pressure analysis

#### 3.10.1 Selection on RSVA/B strains

The entire coding region of the F gene of all African strains (regardless of clade due to the conserved nature of the F gene) were analysed for sites under positive selection. In addition, the South African strains were analysed separately to infer any country-specific selection pressure. In the African RSV-A F African dataset the amino acid position 554 was found to be under positive selection by all models. The country-specific analysis conducted on the RSV-A South African dataset did not identify any selection sites that exhibited positive selection.

For the G gene, multiple sites within the RSV-A African GA2.3.5 dataset were detected under positive selection, specifically, amino acid positions 273, 290, 297, and 314 were consistently identified as undergoing positive selection across all the models used in the analysis. Furthermore, a separate analysis was conducted on the GA2.3.5 South African strains to determine if there were any selection pressures specific to the country. Notably, sites 298, 308, and 314 were identified as being subjected to positive selection, indicating potential evolutionary pressures unique to South Africa.

Positive selection acting on the fusion protein was detected at amino acid position 125 in the RSV-B F African dataset, as indicated by the four selection models employed in the study. A consensus among all the models indicates that amino acid position 4 in the RSV-B F South African dataset was subject to positive selection. The amino acid positions 217, 226, 229, 245, 252, 268, 285, 303, and 310 within the RSV-B GB5.0.5a G gene African dataset, exhibited positive selection across all models. For the South African GB5.0.5a G gene dataset, amino acid positions 217 and 285 were identified under positive selection.

### 3.11 N-linked glycosylation

In the G gene of South African GA2.3.5 strains, residues 85, 103, 135, 237 and 318 were identified as N-glycosylation sites. Residues 85, 103, 135 were highly conserved in COVID-19 era strains and some pre COVID-19 era strains but absent in strains in South Africa in 2018. This may be evidence of selection of strains with these glycosylation sites. The absence of an N-glycosylation site at residue 237 was infrequently detected in strains both prior to and during the COVID-19 era, of note, 8/11 strains sequencing in this study lacked this N-glycosylation site. Residue 318 varied in strains both prior to and during the COVID-19 era.

In the G gene of South African GB5.0.5a strains residues 81, 86, 228, and 294 were identified as frequent N-glycosylation sites. Residues 81 and 86 were more frequently identified as potential N-glycosylation sites in strains that circulated from the COVID-19 era. Residue 228 was identified as a potential N-glycosylation site in a minority of samples mostly from the COVID-19 era including all samples sequencing in this study. In most South African strains, a proline amino acid was observed at this site which prohibits the formation of *N*-linked glycans.

In both the RSV-A and RVS-B F gene African datasets five potential N-glycosylation sites were noted located at residues 27, 70, 116, 126, and 500. These sites were found to be highly conserved in all South African strains regardless of year of detection. The presence of a sixth N-glycosylation site at residue 120 showed more variability. In contrast to the other strains, the study strain SARI-18 displayed an additional distinctive N-glycosylation site at position N437.

## 4 Discussion

To curb the transmission of SARS-CoV-2 during the peak of the COVID-19 pandemic, the government of South Africa declared a national state of disaster on 15 March 2020 with a national lockdown implemented from 27 March to 30 April 2020 with international and local travel restrictions, closure of all non-essential businesses and schools, and citizens were confined to their residences (Bents et al., 2022). Additional non-pharmaceutical interventions including social distancing, travel bans, school closures, and mask wearing were in place between March and November of 2020. These measures impacted the transmission of RSV within South Africa with decreased RSV disease reported in 2020 and the previously established seasonality not observed. Coinciding with the relaxation of NPIs, an out-of-season outbreak of RSV was reported that continued into 2021 (Bents et al., 2022; Perofsky et al., 2022). Disease was reduced between March and November of 2021 as stricter NPIs were implemented to control the third wave of COVID-19 (Bents et al., 2022). This phenomenon was not restricted to South Africa. The global rate of RSV infection decreased dramatically during the peak of the COVID-19 pandemic (Abu-Raya et al., 2023). As NPI measures were relaxed an off-season resurgence of RSV was reported. An example the atypical surge in cases reported during the Australian summer of December 2020 to February 2021 (Britton et al., 2020). Another atypical resurgence of RSV in Senegal characterised by increased RSV detections with a temporal shift was observed between September and October 2022 (Jallow et al., 2022).

This study undertook a whole genome sequencing of both RSV-A and -B strains detected in 2021 and 2022 in the Free State province to understand specific changes in the circulating clades or the emergence of novel strains, if any, associated with the increased disease following the peak of the COVID-19 pandemic. The phylogenetic analysis revealed that over the study period, the circulating strains in South Africa were lineages GA2.3.5 (previously ON1) and GB5.0.5a (previously BA9). Recent studies have corroborated these findings, indicating that GA2.3.5 and GB5.0.5a were the prevailing types circulating prior to and following the COVID-19 pandemic in several countries (Goya et al., 2023; Jallow et al., 2022; Redlberger-Fritz et al., 2023). The RSV-A ON1 lineage has been globally dominant worldwide since its initial detection in Ontario, Canada, in 2010 (Eshaghi et al., 2012; Lu et al., 2019), and during the COVID-19 pandemic (Eden et al., 2022; Jia et al., 2022; Lee et al., 2023). The BA genotype has gained global prevalence since its emergence and is currently the dominant RSV-B genotype in circulation across numerous countries (Gaymard et al., 2018; Trento et al., 2010).

As previously described, in the years immediately prior to the COVID-19 pandemic (2015-2017), ON1 genotype strains were dominant in South Africa (Liu et al., 2020). These variants were frequently introduced into South Africa from diverse geographic origins. During 2020, there was a reduction in the number of GA2.3.5a variants co-circulating in the country evident in both the phylogenetic analysis and in the reconstruction of the relative genetic diversity of GA2.3.5a strains. The strains circulating during the upsurge of disease were largely derived from strains that circulated prior to the COVID-19 era with a small number of variants introduced into the country. The phenomenon of diversification occurring at the local level has been documented and is believed to contribute to the persistence of ON1 variants within a population across various seasons (Agoti et al., 2017; Duvvuri et al., 2015; Lu et al., 2019). This is also in line with what has been observed in other countries where strains that circulated prior to the COVID-19 era were associated with the upsurge in cases once NPIs ceased (Jia et al., 2022; Lee et al., 2022). This contrast with the resurgence of RSV in Argentina in 2021 which was attributed to the introduction of new viral strains from other countries as potential drivers of the outbreak (Dolores et al., 2022).

Of note, the GA2.3.5a strains that persisted from the pre-COVID-19 era in South Africa appear to have given rise to the dominant variant detected in 2021 and 2022 which lacked N-glycosylation residues 85, 103, 135 which were a feature of COVID-19 era strains suggesting a pressure acted on this variant during 2019-2020 selecting strains with these N-glycosylation residues. Interestingly these N-glycosylation residues were detected frequently in strains that circulated in 2013-2015. Residues 298, 308, and 314 were determined to be under positive selection in the South African dataset with residues 273, 290, 297, and 314 in African GA2.3.5. The sites under positive selection were not unique to strains circulating in the COVID-19 era.

In contrast to RSV-A, where the same G-clade had predominated for many years prior to the COVID-19 era, GB5.0.2 circulated as the dominant variant from the mid-2000s to 2015 and GB5.0.5a emerged as dominant from 2018 onwards. The dominant lineage of South African GB5.0.5a strains that circulated prior to the COVID-19 era was largely undetected after 2020. Unlike the GA2.3.5 strains that were largely derived from pre-existing variants, multiple GB5.0.5a variants co-circulated in 2020-2022 with global contemporaneous strains introduced recently into South Africa rather than evolving from endemic variants. For the South African GB5.0.5a G gene dataset, amino acid positions 217 and 285 were identified under positive selection but were not associated with strains emerging in the COVID-19 era. Residues 81 and 86 were more frequently identified as potential N-glycosylation sites in strains that circulated from the COVID-19 era. This is consistent with a previous study (Kamau et al., 2020).

The period of emergence of the GA2.3.5 clade in South Africa was late 2010 and early 2014 for GB5.0.5a. This corresponds with the global emergence of these clades and suggests they were seeding into South Africa soon after their global emergence. The evolutionary estimates in our study differ slightly from previous estimates, 2.033×10^−3^ and 4.081 ×10^−3^ nucleotide substitutions/site/year for GA2.3.5 and GB5.0.5a respectively. The previously described rates of evolution have been for the ON1 lineage rather than the GA2.3.5 clade, with estimates reported of 3.06 × 10^−3^ nucleotide substitutions/site/year for global strains and country-specific analysis of Kenyan isolates reported a rate of 2.89 × 10^−3^ nucleotide substitutions/site/year (Otieno et al., 2016). Similarly, a prior South African study that estimated the rate of evolution for the entire BA lineage reported at 5.8907 × 10^−3^ nucleotide substitutions/site/year (Pretorius et al., 2013).

A decline in the relative genetic diversity was observed in both South African GA2.3.5 and GB5.0.5a viral populations. This bottleneck can be attributed to the reduced circulation of RSV during the peak of the COVID-19 pandemic when NPIs reduced the transmission of respiratory pathogens in the community and numerous variants died out. A subsequent increase in relative diversity could be attributed to increased off-season cases reported as public health measures were eased suggesting the resurgence of RSV variants circulating in the population.

The pandemic-era NPIs created a unique environment where infants lacked exposure to RSV, creating an immunity debt. The increased cohort of immunologically naïve children may have been a contributing factor in selecting which variants flourished during the off-season outbreaks. The glycosylation of RSV is a significant characteristic that determines the antigenicity of the virus and exhibits considerable variation among different strains of RSV (Gimferrer et al., 2015). Notably, the virus can modify the potential N-linked glycosylation sites, resulting in changes to the antigenic properties of the viral protein (Feng et al., 2022). This modification masks specific epitopes from the host's immune system, thereby affecting the virus's interaction with host immunity (Collins et al., 2013). In both the RSV-A and RVS-B F gene datasets the same potential N-glycosylation sites were observed and were highly conserved regardless of the year of detection suggestive of the significance of the sites in the protein function and biological activity (Feng et al., 2022; Leemans et al., 2019; Schobel et al., 2016). The present research extends the previous finding of an absence of an N-glycosylation site at residue 120 in the RSV-A Fusion protein from strains in South Africa that were identified after 2018 (Mabilo et al., 2022). The N-glycosylation for the G protein showed more variability in strains that emerged in the post-COVID-19 era. For GA2.3.5 strains, N-glycosylation of residues 85, 103, and 135, was associated with the emergence of COVID-era strains. N-glycosylation residues 237 and 318 were inconsistently detected in both pre- and COVID-era samples. For South African GB5.0.5a strains residues 81 and 86 were more frequently identified as potential N-glycosylation sites in strains that circulated from the COVID-19 era. The strains from the Free State province exhibited an additional N-glycosylation site at residue 228 that was rarely seen in other strains.

Based on our clinical data, and due to the limited case number and sampling design it appears the risk factors for severe RSV infection identified were few and no significant conclusions could be drawn from the clinical outcomes. Although, the available clinical data of participants in this study mirrors a severe infection, however no conclusive statical analysis could be conducted due to inconsistency of the available clinical data. As such this was one of the significant limitations of the study. Drawing from previous studies coinfections have the potential to heighten the severity of RSV disease, however, a direct correlation between coinfection and disease severity remains inconclusive, as others found no definitive link between disease severity and coinfection (Goka et al., 2014; Luo et al., 2020; Scotta et al., 2016; Tan et al., 2021). In this study, due to the multiplex PCR system employed in the diagnosis of children in the SARI group, co-infection involving other respiratory viruses such as rhinovirus, SARS-CoV-2, and adenovirus were detected. However, children in the respiratory distress arm had no co-infection documented and may be due to the use of a multiplex PCR with limited viral pathogens targeted. In essence, the adoption of a multiplex system in the diagnosis of respiratory infection in children could aid early identification of co-infection which could guide clinical decisions and ultimately result in better clinical outcomes (Lamrani Hanchi et al., 2021).

This study underscores the continued importance of surveillance efforts in detecting and characterizing new variants of RSV to understand the circulating patterns of these strains. The primary limitation of this research was the sample size (samples collected during the pandemic), limited clinical information, and quantity of RSV genomes sequenced in this study. Nevertheless, to the best of our knowledge, this is the first report on the diversity of RSV-A and -B circulating strains during the COVID-19 period characterised by off-season outbreaks of RSV in South Africa.

In conclusion, this study demonstrated that 2020-2021 RSV surge in South Africa during the COVID-19 pandemic was primarily caused by pre-existing lineages rather than any unique or novel genetic variant. The implementation of NPIs for COVID-19 exerted pressure on the transmission dynamics of RSV, resulting in the delay of epidemic seasons in 2020 and an atypical rise in RSV-positive cases during the spring-summer period. Further investigation is necessary to explore the impact of the shift in RSV epidemiology and on potential future outbreaks. Gaining a deeper understanding of the molecular diversity and evolution of RSV through continuous monitoring during pandemic periods is important, it also can offer valuable insights and assist in making informed public health decisions as well as advancing vaccine development.

## Financial support

The research received financial support from the 10.13039/100000865Bill and Melinda Gates Foundation, grant number BMGFOPP1180423_2017 and INV-046917, the 10.13039/100004423World Health Organization (grant number 2023/1387942-0), the National Research Foundation (NRF), South Africa, grant number 120814, the Poliomyelitis Research Foundation (PRF), South Africa, grant number 19/16 plus 23/56 and the South Africa Medical Research Council Self-Initiated Research (SAMRC-SIR) grants; all awarded to MMN.

## Potential conflicts of interest

The authors of the study declare no conflicts of interest.

## Ethics statement

This study was approved by the ethics committee of the University of Free State (HSREC: UFS-HSD2021/1616/2501-0002).

## Supplementary data

Additional materials have been provided to supplement the main content. The genome sequences generated in this study have been submitted to NCBI. The complete whole genomes sequences comprises of this accession numbers PP784969- PP784987, G – gene with the accession PP660597-PP660616 and F – gene accession PP660578-PP660596

## CRediT authorship contribution statement

**Hlengiwe Sondlane:** Writing – original draft, Visualization, Validation, Project administration, Methodology, Investigation, Formal analysis, Data curation. **Ayodeji Ogunbayo:** Writing – review & editing, Validation, Methodology, Formal analysis, Data curation. **Celeste Donato:** Writing – review & editing, Software, Formal analysis, Data curation. **Milton Mogotsi:** Writing – review & editing, Validation, Methodology. **Mathew Esona:** Writing – review & editing, Formal analysis, Data curation. **Ute Hallbauer:** Writing – review & editing, Resources. **Phillip Bester:** Writing – review & editing, Supervision, Resources. **Dominique Goedhals:** Writing – review & editing, Supervision, Resources. **Martin Nyaga:** Writing – review & editing, Supervision, Resources, Project administration, Funding acquisition, Conceptualization.

## Declaration of competing interest

The authors declare that they have no known competing financial interests or personal relationships that could have appeared to influence the work reported in this paper.

## Data Availability

Data will be made available on request.

## Appendix Supplementary materials

Image, application 1
