# Corrigendum to “Whole genome molecular analysis of respiratory syncytial virus pre and during the COVID-19 pandemic in Free State province, South Africa” [Virus Research, Volume 347, September 2024, 199421]

**DOI:** 10.1016/j.virusres.2024.199449

**Published:** 2024-09-17

**Authors:** Hlengiwe Sondlane, Ayodeji Ogunbayo, Celeste Donato, Milton Mogotsi, Mathew Esona, Ute Hallbauer, Phillip Bester, Dominique Goedhals, Martin Nyaga

**Affiliations:** a Next Generation Sequencing Unit and Division of Virology, Faculty of Health Sciences, University of the Free State, Bloemfontein, South Africa; b Enteric Diseases Group, Murdoch Children's Research Institute, Parkville, VIC, Australia; c Department of Paediatrics, The University of Melbourne, Parkville, VIC, Australia; d The Centre for Pathogen Genomics, The Doherty Institute, University of Melbourne, Australia; e Diarrheal Pathogens Research Unit, Sefako Makgatho Health Sciences University, Medunsa 0204, Pretoria, South Africa; f Department of Paediatrics and Child Health, Faculty of Health Sciences, University of the Free State, Bloemfontein, South Africa; g Division of Virology, School of Pathology, Faculty of Health Sciences, University of the Free State, Bloemfontein, South Africa; h PathCare, Pretoria, South Africa

## Abstract

Respiratory syncytial virus (RSV) is the most predominant viral pathogen worldwide in children with lower respiratory tract infections. The coronavirus disease 2019 (COVID-19) pandemic and resulting non-pharmaceutical interventions perturbed the transmission pattern of respiratory pathogens in South Africa. A seasonality shift and RSV resurgence was observed in 2020 and 2021, with several infected children observed.

Conventional RSV-positive nasopharyngeal swabs were collected from various hospitals in the Free State province, Bloemfontein, South Africa, from children suffering from respiratory distress and severe acute respiratory infection between 2020 to 2021. Overlapping genome fragments were amplified and complete genomes were sequenced using the Illumina MiSeq platform. Maximum likelihood phylogenetic and evolutionary analysis were performed on both RSV-A/-B G-genes with published reference sequences from GISAID and GenBank. Our study strains belonged to the RSV-A GA2.3.2 and RSV-B GB5.0.5a clades. The upsurge of RSV was due to pre-existing strains that predominated in South Africa and circulating globally also driving these off-season RSV outbreaks during the COVID-19 pandemic. The variants responsible for the resurgence were phylogenetically related to pre-pandemic strains and could have contributed to the immune debt resulting from pandemic imposed restrictions. The deviation of the RSV season from the usual pattern affected by the COVID-19 pandemic highlights the need for ongoing genomic surveillance and the identification of genetic variants to prevent unforeseen outbreaks in the future.

The authors regret <Several minor grammatical and syntax errors can be seen from introduction, some in methodology and a few in the results section>.

The authors would like to apologise for any inconvenience caused.

## 1 Introduction

Respiratory syncytial virus (RSV) is recognised as the most frequent cause of viral lower respiratory tract infection (LRTI) in infants and children ≤5 years worldwide, and a leading cause of hospitalisation, mortality, and morbidity (Demont et al., 2021; Shi et al., 2017; Weinberg, 2017). RSV infects nearly 90 % of children within the first two years of life, nearly 40 % of whom develop LRTI with the first infection episode (Brady et al., 2014; Meissner, 2016). Also, RSV offers partial immunity due to waning immunological memory, characterized by recurrent infections that may continue into adulthood (Griffiths et al., 2017; Nam and Ison, 2019). Almost 90 % of RSV-associated fatalities are recorded in children ≤5 years from low income (or low resourced) countries (Liu et al., 2020).

In temperate regions, RSV has a distinct seasonal circulation in the late autumn or early winter and in tropical regions infections occur predominantly during the rainy season (Khor et al., 2012). Despite the global impact of RSV on public health, there are currently no licensed RSV vaccines or available effective antivirals to treat acute infection (Feng et al., 2022; Mazur et al., 2018). Palivizumab prophylactic monoclonal antibody (mAb) has been widely used in the prevention of RSV and has proven to be effective against RSV infections in high-risk children (Cadena-Cruz et al., 2023; Elawar et al., 2021). In addition, novel mAb nirsevimab, was recently approved by the FDA for the prevention of RSV infection in infants (Simoes et al., 2023). A new vaccine, known as ABRYSVO, administered to pregnant women, protects infants from RSV associated LRTI. Individuals older than 60 years may also be given the vaccine (Ernst, 2023).

Recently RSV has been reclassified under the genus *Orthopneumovirus,* belonging to the family *Pneumoviridae* and order Mononegavirales (Rima et al., 2017). The genome has approximately 15, 200 nucleotides of single stranded, negative sense RNA encoding 11 viral proteins (Azzari et al., 2021). The virus is classified into RSV-A and RSV-B, the two primary antigenic groups that have been identified as the prevalent strains within the human population (Vandini et al., 2017). The attachment glycoprotein (G) has been extensively employed for RSV genotyping due to its high degree of diversity (Goya et al., 2020; Hibino et al., 2018; Munoz-Escalante et al., 2021). Numerous studies have proposed a unified manner of designating genotypes. Adoption and the implementation of this methodology facilitated the categorization of lineages and sub-genotypes or clades, while simultaneously decreasing the number of identifiable genotypes by almost half in both groups (Chen et al., 2022; Goya et al., 2020). Based on the newly proposed genotype nomenclature, three genotypes were identified and classified within RSV-A, namely GA1, GA2, and GA3, similarly, within RSV-B, seven genotypes were designated as GB1, GB2, GB3, GB4, GB5, GB6 and GB7 (Goya et al., 2020).

Before the COVID-19 pandemic, an estimated 6,000 RSV-associated deaths occurred annually in South Africa (Cohen et al., 2018). The unprecedented COVID-19 pandemic prompted the South African government and other nations to implement nonpharmaceutical interventions (NPIs) to contain the spread of SARS-CoV-2. These NPIs included social distancing, frequent hand washing, travel restrictions, school closures, curfews, and mask wearing (Alanezi et al., 2020; Bents et al., 2022; Ijaz et al., 2022). Although the implemented NPIs were successful in controlling SARS-CoV-2 transmission, they have indirectly disrupted the circulation of other respiratory viruses with a known seasonal pattern, such as RSV, in many countries worldwide (Baker et al., 2020; Tempia et al., 2021; Yeoh et al., 2021; Zheng et al., 2021). Surveillance data from South Africa suggested a significant decline in RSV circulation from the 2020-2021 winter season (Olsen et al., 2021; Tempia et al., 2021). The impact of the NPIs was not uniform amongst the respiratory pathogens, but there was a simultaneous reduction of influenza, and RSV during the COVID-19 pandemic compared to rhinoviruses and respiratory adenoviruses (Abo et al., 2021; Tempia et al., 2021). A delayed RSV season was observed in late 2020 and early 2021 and its resurgence was accompanied by offseason outbreaks following the gradual relaxation of lockdown restrictions (Agha and Avner, 2021; van Summeren et al., 2021). The altered epidemiology and resurgence of RSV was probably due to waned immunity from reduced or lack of exposure to the virus, (Reicherz et al., 2022). This highlights the importance of molecular surveillance to predict potential outbreaks that could have an impact on vulnerable populations.

## 2 Methodology

### 2.1 Study design and patient enrolment

This study utilised samples collected from two existing studies that previously received ethical clearance. Samples were utilised from a paediatric clinical study enrolling children (0-12 years) with respiratory distress requiring hospital admission at Pelonomi Regional Hospital, Universitas Academic Hospital, and National District Hospital in the Free State Province, South Africa. Nasopharyngeal swabs (NPs) were collected and transported for routine testing of RSV, SARS-CoV-2, influenza, parainfluenza, adenovirus and *Bordetella pertussis* at the National Health Laboratory Services (NHLS), Bloemfontein, Free State Province, South Africa. Secondly, another cross-sectional study recruited children who were presenting with severe acute respiratory tract infection (SARI) and required hospitalisation. Patients were recruited from Botshabelo District Hospital, Pelonomi Regional Hospital, and National District Hospital in the Free State Province, South Africa. The World Health Organisation (WHO) definition of SARI was used as criteria for sample collection as described previously (Ogunbayo et al., 2022). Nasopharyngeal swabs (NPs) (BD Diagnostics, Franklin Lakes, NJ, USA) were collected from the children and inserted into a viral transport media (VTM) and placed in ice (4 °C) then transported to the University of the Free State Next Generation Sequencing Unit (UFS-NGS Unit) and were stored at -80 °C until processed. Subsequently, the NPs were tested for several respiratory viruses including RSV as described previously (Ogunbayo et al., 2022). Upon admission, clinicians and or nurses collected relevant clinical and demographic information. Study numbers were used instead of patient identification information.

### 2.3 Sample collection

Samples and data collected from children who were enrolled in the main studies between July 2020 to July 2021 were retrospectively selected. The NPs that were identified as positive for RSV were selected in children ≤5 years at the time of sampling and samples collected during the COVID-19 pandemic. These samples were retrieved from NHLS (n=50) and UFS-NGS Unit (n=19) archives along with the provision of cycle threshold (Ct) values and the list of detected viruses on the respiratory panels.

### 2.4 RNA extraction and cDNA synthesis

Viral RNA was extracted directly from 250 μL of RSV-positive clinical sample using the QIAamp Viral RNA mini kit (QIAgen, Hilden, Germany), according to the manufacturer's recommendations, except for the use of a carrier RNA. RNA eluted in 50 μL of elution buffer was stored at -20 °C until use. Additionally, samples with low-quality RNA were further extracted using an automated extraction machine Chemagic^Tm^ 360 (PerkinElmer, Waltham, MA, USA), according to the manufacturer's recommendations.

### 2.6 Data analysis

#### 2.6.1 Genome assembly

Raw pair-end sequence reads were analysed using Genome Detective, an online based bioinformatics tool (https://www.genomedetective.com/, Accessed 22 November 2022) (Vilsker et al., 2019). The RSV subtypes were assigned, and genome coverage was generated for each sample during the assembly process. Sequences with over 99 % genome coverage were further inspected and analysed using Geneious Prime® version 2022.0.1 (Kearse et al., 2012). The reads were assembled/mapped to a reference sequence for both RSVA and B (Accession number NC_013235 and NC_001781), respectively, using Geneious Prime®, and generated a consensus sequence for each sample.

## 3 Results

### 3.1 Patient demographics and clinical characteristics

Participants were enrolled between 2020 to 2021 and a total of 69 samples were collected. The demographic profiles among the participants were similar, comparable, and unbiased in both groups with respiratory distress and SARI. Among the RSV positive patients, 47/69 (68 %) had LRTI, and 11/69 (16 %) had URTI. The remaining 16 % had no clinical data available. The clinical characteristics of children are shown in Table 1.1. Detailed demographics of the participants of which samples were subsequently used in the analysis are represented in the supplementary material Table 1S1-1S2.

**Table 1.1:** Clinical Characteristics of Patients infected with RSV during the COVID-19 Pandemic

Patient demographics	Paediatric clinical study	Viral metagenomic cross sectional study
**RSV infection cases**	66% ≤1 year	37% ≤ 1year
**Gender** **Males** **Females** **Unspecified**	48% (24/50) 36% (18/50) 16% (8/50)	63% (12/19) 37% (7/19) -
**Suspected diagnosis** **LRTI** **URTI** **Unspecified**	78% (39/50) 2% (1/50) 16% (8/50)	37% (7/19) 47% (9/19) 16% (3/19)
**PICU admission** **Unspecified**	8% (4/50) 16% (8/50)	None 5 % (1/19)
**Feeding difficulty**	-	32% (6/19)
**HIV infected** **Uninfected** **Exposed** **Unspecified**	4% (2/50) 58% (29/50) 22% (11/50) 16% (8/50)	11% (2/19) 89% (17/19) - -
**Chest indrawing**	-	47% (9/19)
**Household member smoking** **Unspecified** **Unknown**	16% (8/50) 16% (8/50) 2% (1/50)	16% (3/19) - -

### 3.4 South African RSV positive cases before and during the COVID-19 pandemic

In South Africa, the first case of COVID-19 was reported on March 5, 2020, and a nationwide lockdown was subsequently imposed on March 27, 2020. The implementation of NPIs during the COVID-19 pandemic, such as travel restrictions, mask-wearing, social distancing, and lockdowns, had initially led to a decrease in the transmission of various respiratory viruses including RSV. Following the relaxation of the implemented NPIs, there were notable summer outbreaks of RSV in South Africa in late 2020. The epidemiological data of RSV before and during the COVID-19 pandemic in South Africa from both inpatients and out-patients from the National Institute of Communicable Diseases (NICD) were examined from January 2016 to December 2022 (Fig. 1.2). Prior to 2020, RSV activity typically began in mid-autumn and persisted throughout the winter months with a peak in cases during winter. However, the easing of these restrictions resulted in a resurgence of RSV cases, which had been relatively low for over a year. During the COVID-19 pandemic, the absence of RSV during winter led to the emergence of offseason outbreaks, marking a significant shift from the usual seasonal distribution of the virus. This is evident from the laboratory-confirmed RSV positive cases reported in 2020, which were associated with an offseason outbreak in summer (Fig. 1.2).

### 3.6 Maximum likelihood phylogenetic tree of RSV-A

In the pre-COVID-19 era, specifically, from 2015 to 2018, there were several co-circulating variants of GA2.3.5 in South Africa, showing high genetic similarity to global strains, primarily from Europe, North America, and other countries within the African continent (Fig. 1.5). The phylogenetic tree showed minor endemic circulation of strains within Africa reflecting the continued introduction of variants from outside the continent. Several variants that circulated in the pre-COVID-19 era were not detected from 2018 onwards including the dominant South African variant that circulated between 2015-2018 which was also detected in Mozambique.

The strains detected in South Africa between 2020-2021 were genetically diverse, forming a number of small clusters and most of these small clusters were closely related to South African strains detected in 2018, suggesting that the increase in RSV-A detected was not due to a dominant new variant recently introduced into South Africa, rather the population was largely derived from strains that had likely evolved within the country over the intervening years. A small number of variants appeared to be recent introductions following the re-opening of borders showing a high degree of genetic similarity to globally circulating strains. No unique clustering patterns were observed with strains from other regions that could suggest sustained introductions of novel variants into the population. The samples from the Free State formed two discrete clusters, with the largest cluster forming a monophyletic cluster with contemporary South African strains from other provinces and most closely related to samples from the Gauteng province. Based on the phylogeny, this variant was likely derived from viruses that circulated in Kenya between 2015-2018. There was a high degree of circulation of all variants across the country indicating the widespread nationwide

### 3.9 The relative genetic diversity of the GA2.3.5 and GB5.0.5a strains

Between 2017 and 2022, the South African GA2.3.5 and GB5.0.5a strains showed very similar patterns of genetic diversity with a slight bottleneck observed between 2019 and 2020 which represented multiple variants ceasing to circulate in the population during the early years of the COVID-19 era coinciding with lockdowns and extensive NPIs. This was followed by an increase in diversity between 2021 and 2022 as multiple variants co-circulated with the introduction of new variants, with diversity decreasing throughout 2022 due to reduced sampling (Fig. 1.7).

## 4 Discussion

To curb the transmission of SARS-CoV-2 during the peak of the COVID-19 pandemic, the government of South Africa declared a national state of disaster on 15 March 2020 with a national lockdown implemented from 27 March to 30 April 2020 with international and local travel restrictions, closure of all non-essential businesses and schools, and citizens were confined to their residences (Bents et al., 2022). Additional NPIs including social distancing, travel bans, school closures, and mask wearing were in place between March and November of 2020. These measures impacted the transmission of RSV within South Africa with decreased RSV disease reported in 2020 and the previously established seasonality not observed.

