# Supplementary material for: Whole genome molecular analysis of respiratory syncytial virus pre and during the Covid-19 pandemic in free state province, South Africa
Source: Virus Res. 2024 Jul 5;347:199421. doi: 10.1016/j.virusres.2024.199421 (PMC11283024; doi:10.1016/j.virusres.2024.199421)
Supplement: Supplementary file 1 [file mmc1.docx]

**Supplementary Table 1S1:** Clinical information for children under the age of ≤5 years recruited for the Paediatric clinical study.

| **Sample ID** | **Age months** | **Gender** | **Symptoms** | **Suspected diagnosis** | **Adm_PICU** | **HIV_Status** | **Pas_Smoking_Ex** | **Duration of stay (days)** | **Ct Values** |
| --- | --- | --- | --- | --- | --- | --- | --- | --- | --- |
| RD-1 | 72 | Male | Cough  Shortness of breath  Sore throat | URTI croup | No | Uninfected | Yes |  | 24,33 |
| RD-2 | 7 | Female | Cough  Fever >38 °C Shortness of breath Nausea/Vomiting General weakness | LRTI | No | Uninfected | No | 12 | 30,84 |
| RD-3 | NA | Male | Cough  Fever >38 °C Shortness of breath Nausea/Vomiting | LRTI | No | Exposed uninfected | Yes | 5 | 20,91 |
| RD-4 | 2 | Female | Cough  Fever >38 °C Shortness of breath | LRTI | No | Exposed uninfected | No | 4 | 28,5 |
| RD-5 | 46 | Male | Cough  Fever >38 °C Nausea/Vomiting | LRTI | No | Uninfected | No | 3 | 23,46 |
| RD-6 | 18 | Male | Cough  Shortness of breath Nausea/Vomiting Diarrhoea | LRTI | No | Uninfected | Yes | 2 | 25,61 |
| RD-7 | <1 | Male | Cough  Fever >38 °C Shortness of breath  Other | Other | Yes | Uninfected | No | 16 | 36,37 |
| RD-8 | 30 | Male | Cough  Sore throat Nausea/Vomiting Irritability/confusion | LRTI | No | Uninfected | Yes | 2 | 19,49 |
| RD-9 | 6 | Male | Cough  Shortness of breath | LRTI | No | Unknown | No | 4 | 18,09 |
| RD-10 | 12 | Male | Cough  Fever >38 °C  Shortness of breath  Diarrhoea Irritability/confusion | LRTI | No | Uninfected | No | 5 | 21,23 |
| RD-11 | 4 | Male | Cough  Fever >38 °C  Shortness of breath Nausea/Vomiting | LRTI | No | Uninfected | Yes | 3 | 27,91 |
| RD-12 | 1 | Female | Cough | LRTI | No | Uninfected | No | 0 | 15,15 |
| RD-13 | 2 | Female | Cough  Shortness of breath Irritability/confusion | LRTI | No | Uninfected | No | 2 | 19,92 |
| RD-14 | 9 | Male | Cough  Fever >38 °C  Nausea/Vomiting | LRTI | No | Infected on ART | No | 1 | 29,49 |
| RD-15 | 11 | Female | Cough  Fever >38 °C  Shortness of breath | LRTI | No | Exposed uninfected | No | 12 | 21,52 |
| RD-16 | 1 4 | Female | Cough  Fever >38 °C  Shortness of breath | LRTI | No | Uninfected | No |  | 32,6 |
| RD-17 | 1 | Male | Cough  Fever >38 °C  Shortness of breath Irritability/confusion  General weakness | LRTI | No | Uninfected | No | 4 | 30,18 |
| RD-18 | 1 | Male | Cough  Fever >38 °C  Shortness of breath | LRTI | No | Uninfected | No | 13 | 25,08 |
| RD-19 | 3 | Female | Cough  Shortness of breath | LRTI | No | Uninfected | Yes | 2 | 20,07 |
| RD-20 | 1 | Male | Cough  Nausea/Vomiting | LRTI | No | Uninfected | No | 3 | 21,62 |
| RD-21 | 1 | Male | Cough  Shortness of breath Irritability/confusion  General weakness | - | No | Uninfected | Yes | 5 | 26,38 |
| RD-22 | 2 | Male | Cough  Shortness of breath  Diarrhoea  Nausea/Vomiting | LRTI | No | Uninfected | No | 2 | 28,88 |
| RD-23 | 4 | Female | Cough  Shortness of breath | LRTI | No | Exposed uninfected | No | 5 | - |
| RD-24 | 1 | Male | Shortness of breath Irritability/confusion | LRTI | No | Uninfected | No | 7 | - |
| RD-25 | NA | Male | Cough | LRTI | Yes | Infected on ART | No | 30 | - |
| RD-26 | 3 | Female | Cough | LRTI | Yes | Exposed uninfected | No | 10 | - |
| RD-27 | 12 | Male | Cough | LRTI | No | Exposed uninfected | No |  | - |
| RD-28 | 3 | Male | Cough  Fever >38 °C  Shortness of breath Nausea/Vomiting Diarrhoea Irritability/confusion | LRTI | No | Uninfected | No | 7 | - |
| RD-29 | 11 | Female | Cough  Fever >38 °C  Shortness of breath | LRTI | No | Uninfected | No | 5 | - |
| RD-30 | 5 | Male | Cough  Shortness of breath | LRTI | No | Uninfected | No | 12 | - |
| RD-31 | 37 | Female | Cough  Fever >38 °C | LRTI | No | Exposed uninfected | Unknown | 3 | - |
| RD-32 | 12 | Female | Cough  Fever >38 °C  Shortness of breath Nausea/Vomiting  Diarrhoea  General weakness | LRTI | No | Exposed uninfected | No |  | 25,11 |
| RD-33 | <1 | Female | Cough  Fever >38 °C | LRTI | No | Exposed uninfected | No | 5 | 31,12 |
| RD-34 | 30 | Female | Cough  Fever >38 °C  Myalgia  Nausea/Vomiting Irritability/confusion  General weakness | LRTI | No | Uninfected | No | 1 | 22,81 |
| RD-35 | 1 | Male | Cough  Shortness of breath Nausea/Vomiting | LRTI | No | Uninfected | No | 3 | - |
| RD-36 | 44 | Male | Cough  Shortness of breath | LRTI | No | Uninfected | Yes | 3 | - |
| RD-37 | 4 | Female | Cough  Shortness of breath | LRTI | No | Uninfected | No | 6 | - |
| RD-38 | 3 | Female | Cough  Shortness of breath Nausea/Vomiting | LRTI | Yes | Uninfected | No | 13 | - |
| RD-39 | 2 | Female | Cough  Shortness of breath Irritability/confusion | LRTI | No | Exposed uninfected | No | 4 | 24,7 |
| RD-40 | 11 | Male | Cough  Fever >38 °C  Shortness of breath  Myalgia  Diarrhoea Irritability/confusion | LRTI | No | Uninfected | No | 4 | 24,1 |
| RD-41 | 3 | Female | Cough  Fever >38 °C  Shortness of breath Other | LRTI | No | Exposed uninfected | No | 4 | 31,84 |
| RD-42 | 47 | Male | Cough  Fever >38 °C  Diarrhoea  General weakness | LRTI | No | Uninfected | No | 7 | 31,61 |

**Supplementary Table 1S2:** Clinical information for children under the age of ≤5 years recruited for the Metagenomics cross sectional study

| **Sample synonymous no** | **Age** | **Gender** | **Symptoms** | **Suspected diagnoses** | **ICU admission** | **HIV infected** | **Household member smoking** | **Feeding difficulty** | **Chest indrawing** | **Need for oxygen** | **Creche attendance** | **Viruses detected (coinfections)** | **RSV**  **ct values** |
| --- | --- | --- | --- | --- | --- | --- | --- | --- | --- | --- | --- | --- | --- |
| SARI-6 | 4 months | Male | Fever ≥ 38° C, Cough, Difficulty in breathing | URTI (Pneumonia) | No | Yes | No | No | Yes | Yes | No | - | 26.2 |
| SARI-15 | 6 Months | Male | Fever ≥ 38° C, Cough, Difficulty in breathing | URTI (Pneumonia) | No | Yes | No | No | Yes | Yes | No | - | 25.6 |
| SARI-16 | 3 Years | Female | Fever ≥ 38° C, Cough, Wheezing | LRTI (Pneumonia) | No | No | No | No | No | No | No | Adenovirus (33.4) | 22.5 |
| SARI-17 | 2 weeks | Female | Fever ≥ 38° C, Cough, Difficulty in breathing | URTI (Pneumonia) | No | No | No | Yes | Yes | Yes | No | - | 25.9 |
| SARI-18 | 3 years 2 months | Female | Fever ≥ 38° C, Cough, Severe Pneumonia | URTI (Pneumonia) | No | No | No | No | Yes | Yes | No | Rhinovirus/Enterovirus (30.9) | 28.2 |
| SARI-19 | 2 years 2 months | Female | Fever ≥ 38° C, Cough, Difficulty in breathing | LRTI (Pneumonia) | No | No | No | no | Yes | Yes | No | Human metapneumovirus (34.3) | 26.5 |
| SARI-20 | 1 year 2 months | Female | Fever ≥ 38° C, Cough, wheezing | URTI (Pneumonia) | No | No | No | Yes | No | Yes | No | Rhinovirus/Enterovirus (35.2) | 26.3 |
| SARI-21 | 10 months | Male | Fever ≥ 38° C, Cough, Difficulty in breathing, wheezing | LRTI (Bronchiolitis) | No | No | Yes | yes | No | Yes | Yes | Rhinovirs/Enterovirus (31.0) | 22.8 |
| SARI-22 | 8 Months | Male | Fever ≥ 38° C, Cough, Difficulty in breathing | LRTI (Pneumonia) | No | No | No | Yes | Yes | Yes | No | Rhinovirus (29.1) | 20.8 |
| SARI-23 | 2 Years | Male | Fever ≥ 38° C, wheezing | URTI (Pneumonia) | No | No | No | No | No | No | No | Coronavirus NL63 (32.0) | 20.7 |
| SARI-24 | 1 year 4 months | Male | Fever ≥ 38° C, Cough, Difficulty in breathing | URTI (Pneumonia) | No | No | Yes | No | Yes | Yes | Yes | - | 21.3 |
| SARI-25 | 2 years 3 Months | Male | Fever ≥ 38° C, Cough | URTI (Pneumonia) | No | No | No | No | No | No | No | Parainfluenza virus 3 (31.0), Rhinovirus (32.0) | 22.5 |
| SARI-26 | 1 year 9 months | Male | Fever ≥ 38° C, Cough, Difficulty in breathing | LRTI | No | No | Yes | No | No | No | No | - | 26.2 |
| SARI-27 | 1 year 1 months | Female | Fever ≥ 38° C, Cough, Difficulty in breathing | URTI (Pneumonia) | No | No | No | Yes | Yes | Yes | No | Parainfluenza virus 3(31.7), Rhinovirus/Enterovirus (33.3), | 34.9 |
| SARI-30 | 5 months | Male | Fever ≥ 38° C, Cough, Difficulty in breathing, wheezing | Unspecified (Pneumonia) | No | No | No | No | Yes | Yes | No | - | 20.3 |
| SARI-31 | 8 months | Female | Fever ≥ 38° C, Cough, Difficulty in breathing | LRTI (Pneumonia) | unspecified | No | No | No | No | No | No | - | 15.3 |
| SARI-34 | 1 year 3 months | Male | Fever ≥ 38° C, Cough, Wheezing | LRTI (Broncholitis) | No | No | No | no | Yes | Yes | No | Rhinovirus/Enterovirus (34.3), SARS-CoV-2 (35.5) | 24.1 |
| SARI-35 | 3 years 8 months | Male | Fever ≥ 38° C, Cough | Unspecified (Broncholitis) | No | No | No | No | No | No | No | Rhinovirus (32.9) | 25.4 |
| SARI-36 | 1 year 3 months | Male | Fever ≥ 38° C, Cough | Unspecified | No | No | No | Yes | No | No | Unspecified | Adenovirus (34.1) | 26.6 |

| **Sample** | **Subgroup** | **Reads**  **Mapped**  **to reference** | **Coverage** | **Depth** | **Length** | **Viruses detected** | **Coinfection detected by QIAstat panel** | **Cycle threshold (Ct)** |
| --- | --- | --- | --- | --- | --- | --- | --- | --- |
| **Participants** |  | | | | | | | |
| hRSV/A/ZAF/UFS-NGS UNIT/RD-3\| 2021-02-24 | A | 210328 | 84.2 | 1716.1 | 12815 | RSV | - | 20,91 |
| hRSV/A/ZAF/UFS-NGS UNIT/RD-4\|2021-02-28 | A | 272038 | 100 | 1923.9 | 15218 | RSV | - | 28,5 |
| hRSV/A/ZAF/UFS-NGS UNIT/RD-5\| 2021-03-11 | A | 209278 | 100 | 1534.8 | 15220 | RSV | - | 23,46 |
| hRSV/B/ZAF/UFS-NGS UNIT/SARI-6\|2021-02-11 | B | 344108 | 100 | 2373.8 | 15219 | RSV | - | 26.2 |
| hRSV/A/ZAF/UFS-NGS UNIT/RD-8\| 2021-02-21 | A | 224844 | 100 | 1560.8 | 15217 | RSV | - | 19,49 |
| hRSV/A/ZAF/UFS-NGS UNIT/RD-9\| 2021-02-23 | A | 115064 | 99.7 | 797.3 | 15170 | RSV | - | 18,09 |
| hRSV/A/ZAF/UFS-NGS UNIT/RD-10\|2021-03-16 | A | 130005 | 99.8 | 1568.9 | 15180 | RSV | - | 21,23 |
| hRSV/B/ZAF/UFS-NGS UNIT/RD-11\|2021-05-04 | B | 174056 | 88.9 | 1361.3 | 13539 | RSV | - | 27,91 |
| hRSV/B/ZAF/UFS-NGS UNIT/RD-12\|2021-03-16 | B | 241883 | 87.5 | 1997.8 | 13319 | RSV | - | 15,15 |
| hRSV/A/ZAF/UFS-NGS UNIT/RD-13\| 2021-03-14 | A | 215118 | 100 | 1535.7 | 15216 | RSV | - | 19,92 |
| hRSV/A/ZAF/UFS-NGS UNIT/RD-14\| 2021-03-15 | A | 200327 | 85.2 | 1629.8 | 12976 | RSV | - | 29,49 |
| hRSV/B/ZAF/UFS-NGS UNIT/SARI-15\|2021-06-16 | B | 164869 | 97.7 | 1230.4 | 14876 | RSV | - | 25.6 |
| hRSV/A/ZAF/UFS-NGS UNIT/SARI-16\|2020-12-21 | A | 138328 | 100 | 1005.7 | 15216 | RSV | Adenovirus (33.4) | 22.5 |
| hRSV/B/ZAF/UFS-NGS UNIT/SARI-17\|2020-21-12 | B | 363351 | 99.9 | 2605.1 | 15204 | RSV | RSV A+B (25.9) | 25.9 |
| hRSV/B/ZAF/UFS-NGS UNIT/SARI-18\|2020-12-02 | B | 232861 | 98.7 | 1503.5 | 15025 | RSV | Rhinovirus/Enterovirus (30.9) | 28.2 |
| hRSV/B/ZAF/UFS-NGS UNIT/SARI-19\|2020-12-29 | B | 252485 | 99.9 | 1743.7 | 15204 | RSV | Human metapneumovirus (34.3) | 26.5 |
| hRSV/A/ZAF/UFS-NGS UNIT/SARI-30\| 2021-01-05 | A | 358164 | 100 | 2509.1 | 15221 | RSV | - | 20.3 |
| hRSV/B/ZAF/UFS-NGS UNIT/SARI-31\|2021-02-11 | B | 361250 | 99.5 | 2672 | 15142 | RSV | - | 15.3 |
| hRSV/B/ZAF/UFS-NGS UNIT/SARI-34\|2021-03-02 | B | 226421 | 99.9 | 1711.8 | 15212 | RSV | Rhinovirus/Enterovirus (34.3), SARS-CoV-2 (35.5) | 24.1 |
| hRSV/A/ZAF/UFS-NGS UNIT/SARI-35\|2021-03-02 | A | 447648 | 100 | 3458.3 | 15221 | RSV | Rhinovirus (32.9) | 25.4 |
| hRSV/B/ZAF/UFS-NGS UNIT/SARI-36\|2020-12-14 | B | 602006 | 99.9 | 4629.3 | 15217 | RSV | RSV A+B (26.6), Adenovirus (34.1) | 26.6 |

**Supplementary Table 1S3:** Genome coverage assigned subtype and the length of RSV-A and RSV-B sequences analysed.

**Supplementary table 1S4:** Nucleotide coverage % for each of the RSV coding regions and protein against the reference strain

| **Sample** | **NS1** | **NS2** | **N** | **P** | **M** | **SH** | **G** | **F** | **M2-1** | **M2-2** | **L** |
| --- | --- | --- | --- | --- | --- | --- | --- | --- | --- | --- | --- |
| hRSV/A/ZAF/UFS-NGS UNIT/RD-3\| 2021-02-24 | 78.6% | - | 49.0% | 28.5% | 52.9% | 100% | 100% | 100% | 100% | 100% | 100% |
| hRSV/A/ZAF/UFS-NGS UNIT/RD-4\|2021-02-28 | 100 | 100% | 100% | 100% | 100% | 100% | 100% | 100% | 100% | 100% | 100% |
| hRSV/A/ZAF/UFS-NGS UNIT/RD-5\| 2021-03-11 | 100% | 100% | 100% | 100% | 100% | 100% | 100% | 100% | 100% | 100% | 100% |
| hRSV/B/ZAF/UFS-NGS UNIT/SARI-6\|2021-02-11 | 100% | 100% | 100% | 100% | 100% | 100% | 100% | 100% | 100% | 100% | 100% |
| hRSV/A/ZAF/UFS-NGS UNIT/RD-8\| 2021-02-21 | 100% | 100% | 100% | 100% | 100% | 100% | 100% | 100% | 100% | 100% | 100% |
| hRSV/A/ZAF/UFS-NGS UNIT/RD-9\| 2021-02-23 | 100% | 100% | 100% | 100% | 100% | 100% | 100% | 100% | 100% | 100% | 100% |
| hRSV/A/ZAF/UFS-NGS UNIT/RD-10\|2021-03-16 | 100% | 100% | 100% | 100% | 100% | 100% | 100% | 100% | 100% | 100% | 100% |
| hRSV/B/ZAF/UFS-NGS UNIT/RD-11\|2021-05-04 | 100% | 100% | 100% | 100% | 100% | 100% | 1.7 | 58.1 | 100% | 100% | 100% |
| hRSV/B/ZAF/UFS-NGS UNIT/RD-12\|2021-03-16 | 69.3 | 59.2 | 62.5 | 60.3 | 72.4 | 27.3 | 85.7 | 99.8 | 100% | 100% | 100% |
| hRSV/A/ZAF/UFS-NGS UNIT/RD-13\| 2021-03-14 | 100% | 100% | 100% | 100% | 100% | 100% | 100% | 100% | 100% | 100% | 100% |
| hRSV/A/ZAF/UFS-NGS UNIT/RD-14\| 2021-03-15 | 100% | 100% | 100% | 100% | 100% | 100% | 100% | 100% | 100% | 73.3 | 72.6 |
| hRSV/B/ZAF/UFS-NGS UNIT/SARI-15\|2021-06-16 | 100% | 100% | 100% | 100% | 100% | 100% | 73.7 | 95.8 | 100% | 100% | 100% |
| hRSV/A/ZAF/UFS-NGS UNIT/SARI-16\|2020-12-21 | 100% | 100% | 100% | 100% | 100% | 100% | 100% | 100% | 100% | 100% | 100% |
| hRSV/B/ZAF/UFS-NGS UNIT/SARI-17\|2020-21-12 | 100% | 100% | 100% | 100% | 100% | 100% | 98.3 | 99.3 | 100% | 100% | 100% |
| hRSV/B/ZAF/UFS-NGS UNIT/SARI-18\|2020-12-02 | 100% | 100% | 100% | 100% | 100% | 100% | 78.3 | 100% | 100% | 100% | 100% |
| hRSV/B/ZAF/UFS-NGS UNIT/SARI-19\|2020-12-29 | 100% | 100% | 100% | 100% | 100% | 100% | 98.0 | 99.8 | 100% | 100% | 100% |
| hRSV/A/ZAF/UFS-NGS UNIT/SARI-30\| 2021-01-05 | 100% | 100% | 100% | 100% | 100% | 100% | 100% | 100% | 100% | 100% | 100% |
| hRSV/B/ZAF/UFS-NGS UNIT/SARI-31\|2021-02-11 | 100% | 100% | 100% | 100% | 100% | 100% | 93.3 | 99.8 | 100% | 100% | 100% |
| hRSV/B/ZAF/UFS-NGS UNIT/SARI-34\|2021-03-02 | 100% | 100% | 100% | 100% | 100% | 100% | 99.0 | 99.8 | 100% | 100% | 100% |
| hRSV/A/ZAF/UFS-NGS UNIT/SARI-35\|2021-03-02 | 100% | 100% | 100% | 100% | 100% | 100% | 100% | 100% | 100% | 100% | 100% |
| hRSV/B/ZAF/UFS-NGS UNIT/SARI-36\|2020-12-14 | 100% | 100% | 100% | 100% | 100% | 100% | 100% | 100% | 100% | 100% | 100% |

**Supplementary table 1S5:** Recombination breakpoints across the whole genome derived from RSV-B.

| **RSV-A Sample ID** | **Whole genome recombination events** | | | | | | | | | |
| --- | --- | --- | --- | --- | --- | --- | --- | --- | --- | --- |
|  | Ns1 | NS2 | N | P | M | SH | G | F | M2 | L |
| hRSV/A/ZAF/UFS-NGS-UNIT/SARI-16\|2020-12-21 |  |  | - | - | - | - | - |  |  | - |
| hRSV/A/ZAF/UFS-NGS-UNIT/SARI-30\|2021-01-05 | - |  | - | - | - | - | - | - | - |  |
| hRSV/A/ZAF/UFS-NGS-UNIT/SARI-35\|2021-03-02 |  |  | - | - | - | - | - | - | - |  |
| hRSV/A/ZAF/UFS-NGS-UNIT/RD-14\|2021-03-15 | - | - | - | - | - | - | - | - | - |  |

**Supplementary figure 1S1:** Maximum likelihood phylogenetic tree for RSV-A GA2.3.5 global strains. Each nation is assigned a specific colour. The tips of tree represent the clinical isolates of the infants investigated in this research and the location. Nodes with bootstrap values are represented by black dots.

**Supplementary figure 1S2:** Maximum-likelihood phylogenetic tree for RSV-B GB.5.0.5a global strains. The colours and indications remain consistent with those in Figure S1

**Supplementary figure 1S3:** Maximum credibility clade tree for RSV-A GA2.3.5

**Supplementary figure 1S4:** Maximum credibility clade tree for RSV-B GB5.0.5a.
